# Bioactive constituents of wood rot extract of tea, *Camellia sinensis* L.O. Kuntze against alates of low country live wood termite *Glyptotermes dilatatus* Bugnion and Popoff (Isoptera: Kalotermitidae)

**DOI:** 10.1186/s40064-015-1513-6

**Published:** 2015-11-19

**Authors:** P. D. Senanayake, K. Mohotti, P. A. Paranagama

**Affiliations:** 1 Entomology Division, Tea Research Institute, Talawakelle, Sri Lanka; 2 Department of Chemistry, University of Kelaniya, Kelaniya, Sri Lanka

**Keywords:** *Glyptotermes dilatatus*, *Camellia sinensis*, Semiochemicals, Rotted tea stem, Healthy tea stem

## Abstract

Low country live wood termite (LCLWT), *Glyptotermes dilatatus* is attractive to rotted stumps of tea plant, *Camellia sinensis*. Rotted stumps are formed due to the attack of wood rot fungi in pruned stems. The objective of the present study was to investigate the response of LCLWT to extracts of rotted and healthy stems of susceptible tea cultivars, TRI 2023 and TRI 4042 and tolerant cultivars, TRI 2027 and TRI 4049 and isolate the LCLWT attractive fractions of tea stem extract. Since pieces of rotted stem of both susceptible and tolerant tea cultivars were more attractive to the alates than that of healthy stems, effects of EtOAc extracts of rotted and healthy stems of four tea cultivars were compared on behavior of the alates. The results revealed that the alates positively responded to extracts of rotted tea stems of four tea cultivars than that of healthy tea stems. Therefore hexane, chloroform and aqueous methanol fractions of the extracts of rotted stems were tested against alates using orientation bioassays. Results revealed that the hexane fraction of rotted stem of each cultivar was more attractive than that of the chloroform and methanol fractions. The results of bioassay guided fractionation of the hexane fraction using column chromatography revealed the presence of two bioactive sub-fractions suggesting non-polar compounds in rotted tea wood are more attractive to *G. dilatatus* than other sub-fractions. These two fractions can be used to develop a trapping mechanism to strengthen present IPM program of LCLWT.

## Background

Tea, *Camellia sinensis* growing areas in Sri Lanka are categorized into three agro-ecological regions, according to the elevation, namely low grown (<610 m amsl), medium grown (610–1220 m amsl) and high grown (>1220 m amsl) tea. It has been estimated that the total land area of the low grown tea is 42.2 % of the total tea growing area in the country. The contribution of low grown tea to the national production of made tea is approximately 60 % from the total production (Anonymous 2011). Low country live wood termite (LCLWT) *Glyptotermes dilatatus* Bugnion and Poppof (Isoptera: Kalotermitidae) is an economically important one of the major insect pests of low grown tea plantations in Sri Lanka (Sivapalan and Senaratna 1981). It has been estimated that yield loss due to LCLWT damage was 3000 kg of made tea/ha when 50 % infestation stand for 10 years (Sands 1977).

Tea plant is cultivated for harvesting ‘two leaves and the bud’ and therefore it is pruned periodically to maintain the vegetative growth. Pruned stems undergo die-back and decay due to wood rot fungi such as *Fusarium oxysporum* (Nectriaceae), *F. solani* (Nectriaceae), *Gliocladium roseum* (Bionectriaceae), *Lasiodiplodea theobromae* (Botryosphaeriaceae) and *Myrothecium roridum* (Incertae sedis) (Balasooriya 1998). It has been reported that the alates of *G. dilatatus* initiate the colony in the rotted stump and continue their feeding to the healthy heartwood of the tea plant. Planting of high-yielding tea cultivars have soft-wooded frames (breakable stems or branches of tea plant) of tea bush that suffer extensive die-back and rot after pruning increases the termite infestation and damage (Sivapalan et al. 1981). Of the two types of high yielding tea cultivars, susceptible cultivars are destroyed by LCLWT in about 10 years after pruning while the tolerant cultivars survive about 30–40 years. Nevertheless, tea growers heavily use susceptible cultivars simply due to fact that they produce the high yield (Sivapalan and Senaratna 1981; Zoysa 2008).

Control of this pest is extremely difficult because of their concealed habit (Dantanarayana and Fernando 1970). Current recommendations for managing this pest include planting of tolerant cultivars. Application of fungicidal paint (Bacor 3PA) to protect prune cuts from decay and removal of decayed wood at pruning to remove initial colonies (Anonymous 2003). It was reported that chemical and classical biological control methods are not efficient in the field (Vitarana 1988). Therefore there is a need to explore other potential methods to incorporate present Integrated Pest Management of *G. dilatatus*. There is an increasing interest in the use of semiochemicals for insect pest management. Semiochemicals of tea stem may affect the behavior of LCLWT and potential use of these extracts to control the test insect has not been investigated extensively. Similar studies have been reported to control other economically important pests in agriculture. Blend of volatile compounds of coconut stems and pheromone of Red Palm Weevil, *Rynchophorus ferrugineus* (Coleoptera: Curculionidae) gave efficient control of *R. Ferrugenius* (Gunawardana and Swarnakanthi 1995). Semiochemicals which emit by the decayed wood of the host plant have been investigated by many scientists and confirmed that the decayed wood of the host plant and wood decay fungi play an important role to attract termites. Pine wood decayed by the fungus, *Lenzites* *trabea* Pers. ex. Fr (Gloeophyllaceae) produces attractive compounds to the eastern subterranean termite, *Reticulitermes flavipes* (Kol.) (Isoptera: Rhinotermitidae) (Esenther et al. 1961). Responses of two termite species, *Coptotermes formosanus* Shiraki (Isoptera: Rhinotermitidae), and *Reticulitermes flavipes* (Kol.) (Isoptera: Rhinotermitidae) to three types of wood decay fungi: *Gloeophyllum trabeum* (Pers. ex. Fr.) (Gloeophyllaceae), *Phanerochaete chrysosporium* Burdsall (Phanerochaetaceae) and *Marasmiellus troyanus* (Murrill) Singer (Marasmiaceae), grown in different substrates were reported (Cornelious et al. 2002). Response of termite species to extracts of decayed wood caused by the fungus, *Lenzites trabea* Pers. ex. Fr (Gloeophyllaceae) has been investigated (Allen et al. 1964; Smythe et al. 1967).

In tea cultivation, the tea cultivars are named as ‘resistant’ and ‘susceptible’ cultivars by considering the life time of tea plants and resistant cultivars live more years than the susceptible cultivars. Preliminary laboratory studies have been carried out using volatile and nonvolatile extracts of the rotted wood of tolerant cultivar (TRI 2027) and termite susceptible cultivar (TRI 2023 and TRI 3063) against alates of *G. dilatatus* (Isoptera; Kalotermitidae) and the results indicated that susceptible cultivar TRI 2023 and TRI 2027 were attractive to termites similarly (Samarasinghe et al. 1999). A thorough literature survey on behavioral effects of LCLWT revealed that the behavior of the test insect against two new tea cultivars, TRI 4042 (termite susceptible cultivar) and TRI 4049 (termite tolerant cultivar) have not been studied. Highly susceptible tea cultivar, TRI 3063 which was more attractive than susceptible cultivar TRI 2023, is no longer grown in low country tea plantations. Hence, the present study was aimed to investigate the effect of extracts of rotted and healthy wood of termite tolerant cultivars (TRI 2027 and TRI 4049) and termite susceptible cultivars (TRI 2023 and TRI 4042) on behavior of the alates of *G. dilatatus*. This is the first report on evaluation of effect of TRI 2027, TRI 4049, TRI 2023 and TRI 4042 against the behavior of LCLWT, *G. dilatatus.* The ultimate target of this study is to develop a device to trap LCLWT alates to incorporate present Integrated Pest Management (IPM) program of LCLWT in tea plantations in Sri Lanka.

## Methods

### Insects

*Glyptotermes dilatatus* colonies were collected from termite infested tea bushes at St. Joachim Estate, Rathnapura, (latitude 6°40′58″N and longitude 80°23′57″E and elevation 128 m) from 15th May 2011 to December 2011. Termite colonies in infested stems were maintained in the laboratory in plastic boxes (Ca.12 L) at room temperature (28 ± 2 °C) at saturation conditions (80 % humidity) under 12:12 L:D photoperiod. The insect colonies were maintained to collect alates for bioassays.

### Plant materials

Rotted stems, healthy stems, and leaves from two susceptible tea cultivars namely TRI 2023 and TRI 4042 and two tolerant tea cultivars TRI 2027 and TRI 4049 were collected separately from a chemically untreated tea field of St. Joachim Estate, Ratnapuara. The plant materials were air dried to reduce the moisture content to 14–15 % before transportation and preserved at −5 °C at Entomology research laboratory at TRI, Rathnapura.

### Extraction of rotted and healthy tea stems

Air dried stem of each tea cultivar (500 g) were ground using a laboratory electrical grinder (Wiley Mill). Ground stems were extracted using freshly distilled ethyl acetate (2 L) for 24 h at ambient temperature, with additional stirring using a rotary shaker. The contents were filtered (gravity filtration) and the residue was re-extracted for another 24 h with ethyl acetate (2 L). Combined extracts were evaporated under reduced pressure to a volume of approximately 5 mL. The extracts were transferred into glass vials (7 mL) using a long-drawn Pasture pipette and the solvent was evaporated under gentle stream of nitrogen to dryness. All the vials containing tea stem extracts were kept in a vacuum drying oven (Lab Tech^®^) for 24 h and stored at −5 °C until needed for bioassays, liquid chromatography and chemical analysis (Paranagama et al. 2010).

### Chemical constituents of ethyl acetate extracts of rotted tea stems

Thin layer chromatography (TLC) (aluminum sheets coated with normal phase silica gel 60, F_254_) used to compare the chemical constituents present in the rotted tea stem extracts of four tea cultivars. A small quantity of the extract of each tea cultivar was dissolved in dichloromethane subjected to TLC and developed in a saturated chamber at room temperature with solvent system 2 % MeOH:CH_2_Cl_2_ as the mobile phase. After development, the plate was air dried and sprayed with the *p*-anisaldehyde reagent to the TLC plates to visualize the constituents of each extract (Braz et al. 2012).

### Partition of ethyl acetate extracts of rotted tea stems

Extracts of four tea cultivars were partitioned between hexane (100 mL × 3) and 80 % aqueous MeOH. The aqueous MeOH layer was diluted to 60 % aqueous methanol by the addition of water and partitioned with chloroform (100 mL × 3). Evaporation of hexane, chloroform and aqueous methanol under reduced pressure yielded three fractions. They were transferred into small glass vials for further drying using a stream of nitrogen and vacuum oven for 24 h to remove any remaining solvent. Three fractions obtained from partitioned with hexane containing non polar compounds, CHCl_3_ containing moderate polar compounds and aqueous methanol containing polar compounds were stored separately at −5 °C until used for the bioassays (Paranagama et al. 2010).

### Liquid chromatography for the hexane fraction of the rotted stem extract

A portion of the hexane fraction (438 mg) dried in the vacuum drying oven (Lab Tech^®^) was redissolved in distilled hexane (1 mL).The reconstituted hexane extract was subjected to normal phase liquid column chromatography packed with silica gel (60–120 mesh, 13 g). A mixture of distilled hexane and dichloromethane with increasing amount of dichloromethane was used as elutants to obtain fractions. 2 % MeOH in CH_2_Cl_2_ used as the final elutant to collect polar constitutes in the extract. Fractions (5 mL each) collected were monitored using TLC and fractions that contained similar constituents were combined after observing under the UV lamp (Paranagama et al. 2010).

### Choice chamber bioassay for different parts of the tea plant, against *G. dilatatus*

**Fig. 1:**
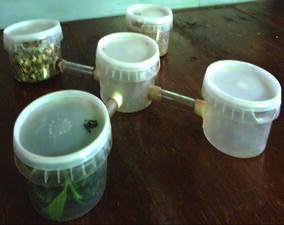
Four arm choice chamber consist of four transparent plastic containers

The choice chamber bioassay was conducted to determine the effects of different parts of fresh tea plant (rotted stems, healthy stems and leaves) on behavior of the termites. Tea cultivars, TRI 2023, TRI 4042, TRI 2027 and TRI 4049 were used in the bioassay. The bioassay was carried out using a four armed choice chamber (Fig. 1). It was consisted of four wide mouthed transparent plastic containers (300 mL) placed at equidistant to each other and connected to the middle transparent container (300 mL) placed in the center of the chamber using glass tubing (diameter 1 cm, length 4 cm). The experimental set up was placed in a plastic box (60.96 × 60.96 × 15.24 cm) and covered with a black paper. Twenty grams (20 g) of pieces of rotted stems, healthy stems, tea leaves of TRI 4042 tea cultivar were introduced into three container separately and the fourth container was used as the control without any plant materials. Twenty undifferentiated alates of *G. dilatatus* just emerged from the tea stems were introduced into the middle container and setup was covered with a black paper. After 14 h, the numbers of alates that moved through the arms into the baited containers were recorded. The experiment was replicated five times. The choice chamber apparatus were thoroughly cleaned using detergent followed by distilled water and dried in subsequent replicates. The above choice chamber bioassay was repeated for rotted stems, healthy stems and tea leaves of TRI 4049, TRI 2023, TRI 2027 separately (Paranagama et al. 2002).

### Olfactometer bioassay for extract of *C. sinensis* against *G. dilatatus*

**Fig. 2:**
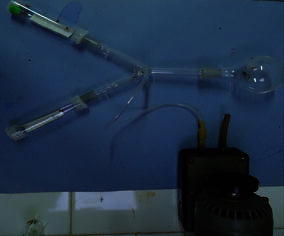
Y′ shaped olfactometer composed of three glass tubes

The behavioral responses of alates of *G. dilatauts* to EtOAC extracts of rotted and healthy stems of *C. sinensis* were investigated in a Y-tube olfactometer (Paranagama et al. 2002; Hoballah et al. 2002). The opening of the intersection of the arms facilitated the air circulation in the olfactometer. The ends of the two tubes of the olfactometer were connected with perforated, glass transparent wide mouthed bottles (20 mL) through the lids and the other end of the Y tube connected to a round bottom flask (100 mL) was used to introduce the test insects (Fig. 2). Two Whatman No. 1 filter papers (1.5 cm × 5 cm), one treated with known amount of the extract dissolved in ethanol and the other treated with equal amount of ethanol were air dried for 10 min to evaporate the solvent. The dried filter papers were placed in the two plastic containers separately and olfactometer placed horizontally on a white background in day light. After switching on the vacuum pump, twenty test insects were introduced into the olfactometer. The number of alates moved into the baited and unbaited containers within 30 min was recorded. At each trial the olfactometer was thoroughly clean with detergent, distilled water and acetone prior to use. Placement of the extract and the ethanol baited filter papers were interchanged randomly in subsequent replicates. Olfactometer was rotated 90° each every 2 min in a clockwise direction to control any directional bias. Test insects, filter paper strips and olfactometer were changed after each replication. All bioassays were carried out between 17 and 19 h. All experiments were replicated five times. In order to select a suitable dose to carry out the bioassays, three doses (0.4, 0.8, 1 mg) of the rotted stem extracts of TRI 4042 were tested separately against alates of *G. dilatatus.* Bioassays were conducted using EtOAC extracts collected from rotted stems, healthy stems of tea cultivars; TRI 2023, TRI 4042, TRI 2027 and TRI 4049. The responses of the alates were investigated with extracts of tea stem given in the following combinations.

1. Rotted vs. healthy stem of TRI 2023.
2. Rotted vs. healthy stem of TRI 2027.
3. Rotted vs. healthy stem of TRI 4042.
4. Rotted and healthy stem of TRI 4049.

To certify that the ethanol has no effect on the behavior of test insect, a bioassay was performed with ethanol treated filter paper strip vs. a filter paper strip without any treatment.

### Unconditional group orientation bio assay

Active extracts to Y-shaped olfactometer bioassays were further fractionated to investigate the active fractions. Unconditional group orientation bioassay was performed for these fractions against alates of *G. dilatatus* (Samarasinghe et al. 1999; Allen et al. 1964). Whatman No. 1 filter paper (14 cm diameter) divided into four sections was placed on a glass Petri dish (14 cm diameter). An appropriate amount of test fractions (hexane fraction, chloroform fraction and aqueous methanol fraction) dissolved in ethanol was applied by a pipette on to three sections and equal volume of ethanol was placed on the fourth section of the filter paper and air dried for 10 min to evaporate the solvent. The dose 100 µg of each fraction were tested in this experiment. Twenty (20) undifferentiated alates were placed in the center of the Petri dish and after 30 min, number of alates in contact with test solution and the solvent treated sections of the filter paper were recorded separately. Bioassay was replicated five times (Samarasinghe et al. 1999). To test the assumption under the null hypothesis of equal distribution of alates of *G. dilatatus* on equivalent filter paper disks, group orientation bioassays were performed. The filter paper in the Petri dish was treated only with the solvent (ethanol) and air dried for 10 min to evaporate the solvent. The number of alates distributed in four sections of the filter paper was recorded separately and compared.

Since the results revealed that hexane fraction was active against the bioassay, the hexane fraction was further fractionated to eight sub-fractions using column chromatography. Sub fractions were labeled as 4042/WR/1/1, 4042/WR/1/2, 4042/WR/1/3, 4042/WR/1/4, 4042/WR/1/5, 4042/WR/1/6, 4042/WR/1/7, 4042/WR/1/8 and the fractions obtained from silica gel column were subjected to group orientation bioassays (Allen et al. 1964; Samarasinghe et al. 1999).

### Statistical analysis

Arcsine transformation was carried out for the data obtained for choice chamber bioassay, unconditional group orientation bioassay and the olfactometer bioassay before performing Two-Way Anova (5 % significance) using SAS version 8 (1999). Mean comparison was performed using Tukey’s Studentized test.

## Results

### Choice chamber bioassay for different parts of the tea plant, against *G. dilatatus*

In order to compare the effect of plant parts (rotted tea stems, healthy tea stems and tea leaves) on behavior of alates, choice chamber bioassays were performed (Table 1). The data analyzed using F test and Tukey’s Studentized test indicates that the plant parts are significantly (F = 142.57, df = 3, P < 0.0001) attracted alates than the untreated control. Mean comparison among rotted stems, healthy stems and tea leaves further showed that the response of alates to rotted stems was significant (P < 0.05). Responses were not differ significantly (F = 0.38, df = 3, P = 0.07681) among cultivars and also between two type of cultivars (tolerant and susceptible) (F = 0.00, df = 1,1, P = 0.9771). There was no significant interaction between cultivars vs plant parts (F = 0.31, df = 3,3, P = 0.9681), resistant/susceptible vs plant parts (F = 0.09, df = 1,3, P = 0.9655).

**Table 1:** Response of alates of *G. dilatatus* to different parts of four tea cultivars, TRI 2023, TRI 4042, TRI 2027 and TRI 4049 of *Camellia sinensis* following choice chamber bioassay

Tea cultivars	% Response (mean ± SE)			
	Rotted stems	Healthy stems	Tea leaves	Untreated control
Susceptible				
TRI 2023	61 ± 5.14^a^	4 ± 1.34^b^	2 ± 0.67^b^	11 ± 1.7^b^
TRI 4042	54 ± 1.78^a^	8 ± 1.78^b^	9 ± 0.89^b^	11 ± 1.34^b^
Tolerant				
TRI 2027	60 ± 5.14^a^	6 ± 1.78^b^	9 ± 1.78^b^	12 ± 2.01^b^
TRI 4049	58 ± 2.23^a^	4 ± 0.67^b^	3 ± 0.89^b^	10 ± 1.5^b^

Each data point represents the mean of five replicates. Twenty insects were used in each experiment. Insects in each container were counted 14 h after introducing the test samples (20 g). Means followed by same letters in each column and same letters in each raw are not significantly different (P < 0.05) according to Tukey’s Studentized test

### Extraction of rotted and healthy tea stems

**Fig. 3:**
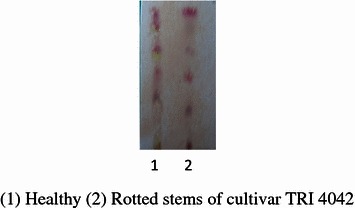
Qualitative analysis of chemical constituents in EtOAc extracts of stems of cultivar TRI 4042 using thin layer chromatography

**Fig. 4:**
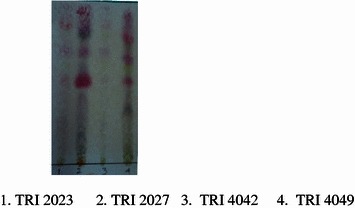
Qualitative analysis of chemical constituents in EtOAc extracts of rotted stems of four tea cultivars using thin layer chromatography

**Fig. 5:**
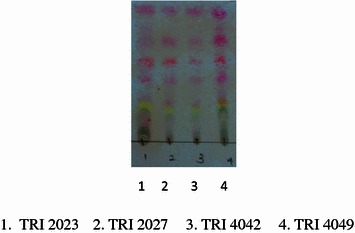
Qualitative analysis of chemical constituents of hexane fraction of rotted stems of tea cultivars using thin layer chromatography

The yields of extracts of rotted and healthy stems of susceptible tea cultivars, TRI 2023, TRI 4042 and tolerant cultivars, TRI 2027 and TRI 4049 are given in Table 2. The yield of each extract of tea cultivar was obtained from 500 g of powdered stems. The yields obtained for the EtOAc extracts were between 412 and 683 mg. The TLC’s was performed to compare the chemical constituents present in extract of TRI 4042 and the results showed that constituents present in the rotted stems were different from that of the healthy stems (Fig. 3). The TLC profiles of the EtOAc extracts of rotted stems demonstrated the presence of similar chemical constituents in the four cultivars (Fig. 4). The weights of fractions obtained after partition of the crude tea stem extract with hexane, chloroform and aqueous methanol are given in Table 2. TLC results of hexane fractions of the four EtOAc extracts also indicated that chemical constituents present in the hexane fraction are similar to each other (Fig. 5).

**Table 2:** Weights of extracts/fractions obtained from rotted and healthy tea stems of susceptible (TRI 2023 and TRI 4042) and tolerant (TRI 2027 and TRI 4049) cultivars

Tea cultivar	EtOAc extract of healthy stems	EtOAc extract of rotted stems	Hexane fraction of rotted stem	Chloroform fraction of rotted stem	Methanol fraction of rotted stem
Weight of extract/fraction (mg)					
TRI 2023	535	547	76.58	257.09	82.05
TRI 2027	616	563	112.6	354.69	84.45
TRI 4042	644	683	122.94	382.9	102.45
TRI 4049	561	412	102.4	247.2	57.12

### Olfactometer bioassay for extract of *C. sinensis* against *G. dilatatus*

Since pieces of rotted stem of four tea cultivars attracted alates of *G. dilatatus*, an attempt was made to study the effect of EtOAc extracts of rotted tea stems obtained from four tea cultivars against behavior of the alates. In order to determine the most preferred dosage of the ethyl acetate extract in the olfactometer bioassays, three doses (0.4, 0.8 and 1 mg) of susceptible tea cultivar, TRI 4042 were tested for the behavioral studies. The results of the bioassays revealed that significantly higher percentage of alates (44 ± 1.4) attracted at dosage of 1 mg (F = 172.0, df = 1, P < 0.0001) while the percentage attracted at dosages of 0.4, 0.8 mg and its respective control were 10 ± 3.3, 23 ± 7.5 and 10.5 ± 3.6, respectively. The Tukey’s studentized test of the results indicated that there was no significant difference found on dosages of 0.4, 0.8 mg and the control (P > 0.05). Hence, the effect of extract/fractions of different tea cultivars on behavior of the alates were studied at dosage of 1 mg in this study.

The effect of extracts from rotted stems vs. healthy stems of each tea cultivar on behavior of alates was compared separately (Table 3). The results showed that response of alates significantly different between extract of rotted stems and extract of healthy stems of four cultivars (F = 252.7, df = −1, P = 0.0001). There is significant difference of response among cultivars (F = 5.89, df = 3, P = 0.0025). Comparison among four cultivars using Tukey’s Studentized Range Test showed that percentage responses of alates to cultivar TRI 2023 (86 ± 1.8) is significantly different (P < 0.05) from TRI 4049 (67 ± 1.7) and TRI 4042 (53 ± 3.6) and not significantly different from resistance cultivar TRI 2027 (80 ± 1.7). There is a significant effect of interaction between cultivar and extract (F = 4.45, df = 3, P = 0.0101). Percentage response of alates to susceptible cultivars was not significantly different from resistant cultivars (F = 0.23, df = 1, P = 0.6321) suggesting that similar constituents may present in extracts of rotted tea stems irrespective of resistant.

**Table 3:** Comparison of behavior of alates of *G. dilatatus* for the fractions of rotted tea stems of susceptible (TRI 2023 and TRI 4042) and tolerant (TRI 2027 and TRI 4049) tea cultivars

Tea cultivars	Percentage response (mean ± SE)			
	Hexane fraction	Chloroform fraction	Methanol fraction	Control (ethanol)
TRI 2023	65 ± 3.5^a^	25 ± 0.9^b^	6 ± 0.9^b,c^	4 ± 0.5^c^
TRI 2027	84 ± 3.6^a^	6 ± 0.5^b^	6 ± 0.5^b,c^	2 ± 0.5^c^
TRI 4042	84 ± 3.8^a^	8 ± 0.8^b^	2 ± 0.4^b,c^	6 ± 1.5^c^
TRI 4049	40 ± 4.1^a^	26 ± 2.6^b^	26 ± 0.7^b,c^	6 ± 0.5^c^

Twenty insects were used in each experiment. Insects in each section were counted 30 min after introducing each extract. Each data point represents the mean of five replicates. The means followed by the similar letters in each column and each raw are not significantly different (p < 0.05) according to Tukey’s mean comparison test

Percentages of test insects responded for extracts of rotted stems of TRI 4042 and TRI 4049 were 53 ± 3.6 and 67 ± 1.7, respectively. Although they were not significantly different (P > 0.05) from each other these values were significantly different (p < 0.05) from the percentage responded (13 ± 2.1 and 15 ± 0.5) to extract of healthy stems of TRI 4042 and TRI 4049 (Table 4).

**Table 4:** Comparison of behavior of alates of *G. dilatatus* for the extract (EtOAc) of rotted and healthy tea stems of susceptible (TRI 2023 and TRI 4042) and tolerant (TRI 2027 and TRI 4049) tea cultivars

Tea cultivars	Percentage responded (mean ± SE)	
	Extract of rotted stems (1 mg)	Extract of healthy stems (1 mg)
Susceptible		
TRI 2023	86 ± 1.8^a^	14 ± 1.1^c^
TRI 4042	53 ± 3.6^b^	13 ± 2.1^c^
Tolerant		
TRI 2027	80 ± 1.7^a^	17 ± 1.3^c^
TRI 4049	67 ± 1.7^a,b^	15 ± 0.5^c^

Twenty insects were used in each experiment. Insects in each container were counted 30 min after introducing each extract. Each data point represents the mean of five replicates. The means followed by the similar letters in each column and each raw are not significantly different (p < 0.05) according to the Tukey’s Studentized Test

### Unconditional group orientation bioassay with fraction

Since EtOAc extracts of rotted tea stems attracted the alates, it was partitioned with hexane, chloroform and aqueous methanol and the response of alates of *G. dilatatus* to each fraction are presented in Table 5. The results showed that the response of alates to the hexane fraction was significantly higher than that of the chloroform fraction and methanol fraction (F = 84.89, df = 3, P < 0.0001) suggesting that hexane fraction contains chemical constituents that attract alates.

**Table 5:** Response of alates of *G. dilatatus* to sub-fractions collected from the hexane fraction of rotted stem extract obtained from the tea cultivar, TRI 4042

Fractions	Weights of fractions (mg)	Percentage response (mean ± SE)
4042/WR/1/2	18	31 ± 1.27^b,c^
4042/WR/1/3	13	50 ± 0.56^a^
4042/WR/1/4	14.4	16 ± 0.57^b,c^
4042/WR/1/5	12	13 ± 0.68^b,c^
4042/WR/1/6	22	17 ± 1.08^b,c^
4042/WR/1/7	32.3	66 ± 1.2^a^
4042/WR/1/8	8.2	5 ± 0.2^c^
Ethanol	–	11 ± 0.46^c^

Twenty insects were used in each experiment. Insects in each sector were counted 30 min after introducing each extract. Each data point represents the mean of five replicates. The means followed by the similar letters in a column are not significantly different (p < 0.05) according to Tukey’s Studentized Test

Responses of alates to the hexane fractions of rotted stem extracts of tolerant cultivars, TRI 2027 (84 %) and TRI 4042 (84 %) were not significantly different (F = 0.07, df = 1, P = 0.9747) from that of susceptible cultivars, TRI 2023 (65 %) and TRI 4049 (40 %) indicating rotted tea stem extracts of four tea cultivars attract the alates. Mean comparison of response of alates to fractions of hexane, chloroform and methanol of the four cultivars showed that response of alates to hexane fraction was significantly higher (TRI2023 = 65 ± 3.5, TRI 2027 = 84 ± 3.6, TRI 4042 = 84 ± 3.8 and TRI 2027 = 40 ± 4.1) than that of chloroform and methanol fractions (P < 0.05). Although response of alates to chloroform faction also significantly higher than that of untreated control and it is not significantly different from methanol fraction (P > 0.05) (Table 3). These results suggest that hexane fraction contains chemical constituents that attract alates.

There was no significant interaction between cultivars vs plant parts (F = 0.31, df = 9, P = 0.9681), resistant/susceptible vs plant parts (F = 0.09, df = 3, P = 0.9655). Responses of alates to different cultivars (F = 0.16, df = 3, P = 0.8551) and resistant vs. susceptible cultivars (F = 0.01, df = 1, P = 0.9113) were not significant. However the interactions between hexane fraction vs resistance/susceptible (F = 3.4, df = 3, P = 0.0230) and hexane fraction vs cultivar (F = 5.96, df = 9, P = 0.0001) were significant. The results obtained for this bioassay is further justified by similar TLC profiles obtained for extracts of the four tea cultivars of rotted tea stems (Fig. 5).

### Evaluation of behavioral effects of sub fractions obtained from the hexane fraction of rotted stem extract cultivar TRI 4042

Since the hexane fractions were more attractive against the alates, it was subjected to column chromatography in order to separate attractive fractions. Eight fractions were obtained and labeled as 4042/WR/1/1–4042/WR/1/8 and Table 5 presents the weights of the sub-fractions and responses of the alates to each sub-fraction. The results revealed that the responses of alates to sub-fractions were significantly different from untreated control (F = 32, df = 7, P < 0.0001) and mean comparison among sub fractions showed that response of alates was significantly attractive to two fractions 4042/WR/1/3 (50 ± 0.56) and 4042/WR/1/7 (66 ± 1.2) suggesting that these two fractions contain semiochemicals that attract alates.

## Discussion and conclusions

In the present study, semiochemicals present in extracts of rotted wood of termite tolerant and susceptible cultivars have been evaluated to develop an eco-friendly pest control agent. Of the extracts of tea cultivars tested in the present study, crude extracts from rotted tea wood from all the test tea cultivars were attractive to termites than extract of the healthy tea wood. Comparison of the extracts of four tea stem cultivars showed that the extracts of rotted wood of TRI 2027 and TRI 2023 were more attractive than that of other tea cultivars used in the experiment. It is suggested that chemical constituents present in extracts of rotted stem of cultivars, TRI 2027 and TRI 2023 may contain the correct percentages of attractive chemicals to attract the alates. Further analysis of the rotted stem extracts indicated that the termites were attracted to the hexane fractions of rotted stems while the chloroform and methanol fractions of rotted wood were not attractive against LCLWT suggesting non-polar, volatile compounds in rotted tea wood attract the alates. These results are in agreement with the preliminary findings in which termite alates were preferred rotted tea stems than that of the healthy tea stems (Sivapalan and Senaratna 1981; Samarasinghe et al. 1999). In a previous research, it has been demonstrated that LCLWT did not respond strongly to extracts of the fungi isolated from the rotted stem, hence combinations of fungal extracts with rotted wood were not used in the present study (Senanayake et al. 2012a, b). However, several studies showed that positive responses by different species of termite for fungus invaded wood over healthy wood (Esenther et al. 1961; Smythe et al. 1967; Cornelious et al. 2002). Extracts of wood decayed by different species of fungi were tested using orientation bioassay and Smythe et al. (1967) reported that the eastern subterranean termite, *Reticulitermes flavipes* showed a strong response for fungal extracts obtained from wood decayed by fungi. Interactions between Formosan subterranean termites and wood decay fungi have been studied and the results indicated that the fungus, *Marasmiellus troyanus* produced a chemical during the process of metabolizing lignin that elicited a positive response in termites (Cornelious et al. 2002).

We confirmed that extracts of rotted tea stem attract LCLWT and bioassay guided fractionation of EtOAc extract obtained from the rotted tea stem indicated hexane fraction contained the attractive compounds for the alates. Two sub-fractions, 4042/WR/1/3 and 4042/WR/1/7 obtained after the column chromatography of the hexane fraction were more attractive to the alates suggesting that bioactive compounds are present in those two fractions. The qualitative analysis of the chemical constituents present in hexane fractions of rotted wood using TLC indicated that chemical constituents present in the hexane fractions of four tea cultivars were similar to each other but different from the healthy stem extracts. However, explanation for higher responses of LCLWT to crude extract obtained from rotted wood of termite susceptible cultivars could be due to the different percentage ratios of chemical constituents present in rotted tea wood. The present study showed that the hexane fraction are responsible for the attractiveness. This is further confirmed as volatile extracts of rotted susceptible cultivars (TRI 2023, TRI 4042) and resistant cultivars (TRI 2027, TRI 4049) were more attractive to alates than that of the healthy stems. Analysis of volatile extracts of rotted stems using gas chromatography–mass spectrometry indicated that the detection of 96 compounds in the four tea cultivars. Among them *n-*hexadecanoic acid and 9,12-octa decadienoic (Z,Z), acid were identified as the major constituents of rotted tea stems (Senanayake et al. 2015). Nevertheless further studies should be carried out by isolating, major constituents in the volatile fraction, n-hexadecanoic acid and 9,12-octa decadienoic (Z,Z) acid and evaluated the effects of the two major compounds against the test insects to confirm the attractiveness. Field studies will be conducted to assess the attractiveness as they can be incorporate into the present IPM of low country live wood termite.
